# Casodex treatment induces hypoxia-related gene expression in the LNCaP prostate cancer progression model

**DOI:** 10.1186/1471-2490-5-5

**Published:** 2005-03-24

**Authors:** Christy A Rothermund, Velliyur K Gopalakrishnan, James D Eudy, Jamboor K Vishwanatha

**Affiliations:** 1 Department of Biochemistry and Molecular Biology, University Nebraska Medical Center, Omaha, Nebraska, USA; 2 Munroe Meyer Center for Human Genetics, University Nebraska Medical Center, Omaha, Nebraska, USA; 3 Neurotoxicology Division, U.S. Environmental Protection Agency, Research Triangle Park, NC 27711, USA; 4 Karpagam Arts and Science College, Coimbatore, India; 5 Department of Molecular Biology and Immunology, Univ. of North Texas Health Science Center, Fort Worth, TX 76107, USA

## Abstract

**Background:**

The changes in gene expression profile as prostate cancer progresses from an androgen-dependent disease to an androgen-independent disease are still largely unknown.

**Methods:**

We examined the gene expression profile in the LNCaP prostate cancer progression model during chronic treatment with Casodex using cDNA microarrays consisting of 2305 randomly chosen genes.

**Results:**

Our studies revealed a representative collection of genes whose expression was differentially regulated in LNCaP cells upon treatment with Casodex. A set of 15 genes were shown to be highly expressed in Casodex-treated LNCaP cells compared to the reference sample. This set of highly expressed genes represents a signature collection unique to prostate cancer since their expression was significantly greater than that of the collective pool of ten cancer cell lines of the reference sample. The highly expressed signature collection included the hypoxia-related genes membrane metallo-endopeptidase (MME), cyclin G2, and Bcl2/adenovirus E1B 19 kDa (BNIP3). Given the roles of these genes in angiogenesis, cell cycle regulation, and apoptosis, we further analyzed their expression and concluded that these genes may be involved in the molecular changes that lead to androgen-independence in prostate cancer.

**Conclusion:**

Our data indicate that one of the mechanisms of Casodex action in prostate cancer cells is induction of hypoxic gene expression.

## Background

Prostate cancer remains the second leading cause of cancer death in men. The progression of prostate cancer from an androgen-dependent, treatable stage to an androgen-independent, non-curable stage is still the primary cause of mortality. Despite screening for early diagnosis, it is still not possible to determine which patients will progress to the androgen-independent stage [1]. Androgen-dependent prostate cancer is responsive to androgen-ablation therapy and the cancer cells die by apoptosis [2]. Androgen ablation or blockage of androgen action through the androgen receptor has been the cornerstone of treatment of advanced prostate cancer [3].

Casodex is a non-steroidal anti-androgen commonly used in the treatment of prostate cancer. Treatment of patients with Casodex has been shown to decrease the progression of early stage prostate cancer to the advanced stages [4]. One of the issues in androgen-blockade therapy is the occurrence of androgen-independent cells which are thought to be the predominant cell type after relapse from therapy. These androgen-independent cells often express mutations in the androgen receptor which accounts for their lack of response to anti-androgens. Recently, it was shown that treatment of patients with Casodex did not result in the occurrence of cells expressing androgen receptor mutations [5]. In our studies, Casodex was chosen since this anti-androgen was shown to act as an androgen receptor antagonist in LNCaP cells. LNCaP cells express a mutation in the hormone binding domain which causes these cells to be stimulated by other anti-androgens [6]. Other studies have shown that Casodex can act as an agonist in prostate cancer [7].

cDNA microarray analysis has become a powerful tool to measure global gene expression changes in both patient biopsy samples and cell culture models. The ability to quantitatively measure differential gene expression provides an opportunity to compare differences in samples which identify potential pathways and modulators of disease processes [8]. A recent study measured differences in gene expression in patient samples and found a clear difference between prostate cancer and benign prostatic hyperplasia [9]. Another study examined differences in gene expression in prostate cancer cell lines and found a unique set of genes whose expression was up regulated [10]. Yet another study showed expression of the enhancer of zeste homologue 2 (EZH2), a polycomb group protein, was overexpressed in androgen-independent prostate cancer and may be a potential modulator of progression [11].

An unusual high tolerance of cancer tissues to low oxygen conditions was one of the first observations regarding cancer cells [12]. The significance of hypoxia in cancer has only recently become a focus of many studies. Recently, it was suggested that expression of hypoxic genes may be responsible for the progression to malignancy in prostate cancer [13]. It was also shown that the more hypoxic a prostate tumor in a patient, the more likely the patient was to experience therapy failure [14]. It has also been suggested that the mechanism of prostate cell death upon androgen withdrawal is due to apoptosis induced by ischemia and hypoxia [15].

In this study we utilized the previously described LNCaP prostate cancer progression model [16,17] to address changes in gene expression that accompany the progression to androgen-independence. Our previous studies showed that chronic treatment of LNCaP-R and LNCaP-UR cells with Casodex selected for cells resistant to apoptosis induction [18]. In the present study, we utilized cDNA microarray analysis to measure the changes in gene expression that had occurred in the LNCaP model after treatment with Casodex to identify potential modulators of prostate cancer progression. We used low passage, androgen-dependent, LNCaP-R cells and high passage, androgen-independent, LNCaP-UR cells and treated cells with Casodex for five weeks to mimic chronic treatment in patients. Our results indicate a set of highly expressed genes that may serve as a signature for prostate cancer progression. A subset of these highly expressed genes were the hypoxia-related genes membrane metallo-endopeptidase (MME), cyclin G2, and Bcl2/adenovirus E1B 19 kD (B-cell lymphoma nineteen kDa interacting protein, BNIP3). This study identifies for the first time the novel pathway of hypoxic gene expression that may modulate the progression of prostate cancer to the androgen-independent state and demonstrate the induction of hypoxia as a mechanism of Casodex action.

## Methods

### Chemicals and reagents

Bicalutamide (Casodex) was provided by AstraZeneca (UK) and was dissolved in fresh dimethylsulfoxide (DMSO) to a concentration of 50 mM, divided into aliquots, and stored at -20°C until further use. Normal human prostate epithelial cell RNA (PrEC) was purchased from Clonetics (Rockland, ME). RPMI 1640, Dulbecco's Modified Essential Media (DMEM), fetal bovine serum (FBS), penicillin-streptomycin (PS), TRIzol, and Hank's Balanced Salt Solution (HBSS) were purchased from Life Technologies (San Diego, CA). Total human reference RNA was purchased from Stratagene (La Jolla, CA).

### Cell culture

LNCaP cells were maintained in RPMI 1640 supplemented with 7% FBS and 1% PS. DU145 and PC3 cells were grown in DMEM supplemented with 5% FBS and 1% PS. Treatment of LNCaP cells with Casodex was performed as follows: Cells were seeded into T75 flasks in RPMI 1640 with 7% FBS and 1% PS and allowed to grow to 70% confluence. The media was then removed and replaced with RPMI 1640 with 7% FBS and 1% PS media containing 1 μM Casodex (vehicle 0.01%). Control cells were fed with media and passaged as needed. Cells grown in media containing Casodex were fed once every three days but did not require passaging during the five-week treatment.

### Isolation of RNA

RNA was isolated from cells using TRIzol according to manufacturer's instructions. Briefly, media were removed and cells were rinsed once in HBSS. A volume of 3 ml of TRIzol was added to each T75 flask and cells were scraped and placed into tubes for chloroform phase separation. RNA was precipitated using isopropanol, rinsed with 70% ethanol in diethylpyrocarbonate water (DEPC), and resuspended in RNase free water. The quality and quantity of RNA was determined using RNA 6000 Nano chips on the Agilent 2100 Bioanalyzer (Agilent Technologies).

### Array fabrication

The slides used in this study were generated as follows: cDNA inserts from Research Genetics Sequence Verified Human cDNA library were amplified using standard procedures in a 96-well format, each well containing 100 μl of PCR reaction mix and 1 μl of bacterial culture as the template. The reaction buffer contained 1.5 mM MgCl_2_, 1× PCR buffer, 0.2 mM dNTPs, 0.25 mM primers, 2.5 units Taq DNA polymerase, and 0.2 % Tween 20. Following an initial denaturation step of 94°C for 60 seconds, 33 cycles of 94°C for 45 seconds, 55°C for 45 seconds, and 72°C for 180 seconds was performed. Verification of the amplified PCR products was performed by agarose gel electrophoresis. Individual PCR products were precipitated overnight at -20°C with sodium acetate (pH 5.2) and ethanol. Following precipitation, the DNA pellets were allowed to dry at room temperature and were resuspended by addition of 30 μl of 3× SSC and 0.1% sarcosyl solution. This procedure resulted in a concentration range of 100 to 499 ng/μl of DNA per sample. The PCR products were spotted 2,305 spots at 375 μm spacing / 2.2 cm^2 ^/ per slide onto poly-L-lysine-coated microscope slides using an Affymetrix GMS 417 arrayer per manufacturer's suggestions at 50% humidity and 25°C. The slides were allowed to dry overnight before the DNA was cross-linked to the slides by UV irradiation.

### Probe synthesis and hybridization

A total of 20 μg RNA from each control and treated samples were reverse transcribed in the presence of amino-allyl modified dUTP [5-(3-aminoallyl)-2'-deoxyuridine 5 triphosphate] and indirectly labeled with cyanine 3 (cy 3) or cyanine 5 (cy 5) dye using the FairPlay microarray labeling kit (Stratagene) according to manufacturers instruction. The absorbance of the samples was measured at 260 nm (for cDNA), 550 nm (for cy 3), 650 nm (for cy 5) to give an indication of reverse transcription and dye labeling efficiency. Cy3 and cy5 labeled samples were mixed with 20 μg human Cot 1 DNA (Stratagene) and 20 μg poly-adenine (Sigma) and the samples were dried in a speed vacuum on medium heat for 2 hours. Dried samples were resuspended in 18 μl of hybridization buffer containing 50% formamide, 5× sodium chloride and sodium citrate (SSC), and 0.1% sodium dodecyl sulfate (SDS). The samples were denatured by incubation at 95°C for 3 minutes then placed on ice for 30 seconds. Slides were prehybridized by incubation for 1 hour at 42°C in prehybridization buffer containing 1.0% bovine serum albumin (BSA), 0.1% SDS, and 5× SSC. The slides were rinsed three times in milli Q water, dipped into 99% isopropanol, and air-dried. Labeled probes were hybridized for 16 hours in a 42°C water bath. Following hybridization, slides were sequentially washed for 5 minutes each in buffers containing; 1× SSC and 0.2% SDS, 0.1× SSC and 0.2% SDS, and 0.1% SSC. Slides were immediately scanned using a GenePix 4000A scanner (Axon Instruments, Union City, CA).

### Data extraction and analysis

Multi image tiff files generated using the GenePix 4000A scanner were imported in GenePix Pro version 4 software (Axon Instruments) for data extraction. Extraction of signal intensity data from each spot on the images was performed and visually checked for accuracy. Raw fluorescent intensities at 532 and 635 nm were measured and local area background was subtracted from each spot using GenePix Pro. Files generated from the extraction were imported in Microsoft Excel for normalization. Trimmed global median normalization was performed on the ratio of medians. For data tables, candidate genes were chosen according to fluorescent intensity readings at 635 nm above 400 units. Normalized ratios of the medians from each of the three hybridizations were averaged and the standard deviation was reported. Normalized, averaged data sets were then analyzed using Cluster analysis and visualized using Treeview [19].

### RT-PCR

RNA transcripts were characterized by RT-PCR using SuperScript II RNase H^- ^Reverse Transcriptase (Invitrogen, Life Technologies) according to manufacturer's instructions. Total RNA (5 μg) isolated from cells using TRIzol was reverse transcribed to cDNA using oligo-dT primers. The PCR product for hypoxia-inducible factor-1α (HIF-1α) was amplified using the following primers: F: 5'-CCTTCGATCAGTTGTCACCA-3' and R: 5'-TGGGTAGGAGATGGAGATGC and gave a 211 bp product. The PCR product for membrane metalloendopeptidase (MME, also referred to as CD10, CALLA, and NEP) was amplified as described by Xiao et. al. [20] and gave a 268 bp product. For a control, β-actin was amplified in parallel using primers; F: 5'-GAAGAGCTACGAGCTGCC-3', and R: 5'-TGATCCACATCTGCTGGA-3' and gave a 368 bp product. Cyclin G2 primers were, F: 5'-AGCCATCAAATGGGGTAGTG-3', and R: 5'-CTTGGGGCAATAGGAATGAA-3' and gave a product of 502 base pairs. BNIP3 primers were; F: 5'-GCTCCTGGGTAGAACTGCAC-3', and R: 5'-TCTTCATGACGCTCGTGTTC-3' and gave a 411 base pair product. The following RT-PCR cycling parameters were used; 42°C for 2 minutes, 42°C for 50 minutes, 70°C for 15 minutes, and hold at 4°C indefinitely. A volume of 5.0 μl of the RT-PCR reaction product was added to each PCR reaction with 1.0 μl each of forward and reverse primers. The PCR cycling parameters used for Cyclin G2 and BNIP3 were; Step 1, 95°C for 3.0 min; Step 2, 94°C for 1 min; Step 3, 60°C for 1 min; Step 4, 72°C for 45 sec; Step 5, repeat steps 2 through 4 thirty-five times; Step 6, 72°C for 3 min; and Step 7, hold at 15°C until further use. The PCR product of VEGF was amplified as described by Munaut *et al *[21], Glut-1 was amplified as described by Ito *et al *[22], and Carbonic anhydrase-IX and XII were amplified as described by Nishimori *et al *[23].

### Real time PCR analysis

Quantitative Real Time PCR reactions were performed using QuantiTect SYBR Green RT-PCR kit (Qiagen Inc., Valencia, CA) as per manufacturer's instructions. Each reaction was set up with 1/10^th ^the volume of cDNA generated by RT-PCR as described above. The PCR cycling parameters used for HIF-1α are initial incubation of 95°C for 15 minutes, 40 cycles of 95°C for 15 seconds, 60°C for 30 seconds, 72°C for 30 seconds, were performed. Reactions, in duplicate, were performed on a BioRad iCycler thermalcycler using iCycler software version 2.3v. The comparative CT equation denotes the expression level of the gene in a given sample, normalized with in the sample to an endogenous reference gene and relative to the expression level of the same gene in another sample can be represented as ΔΔCT= [ΔCT_(sampleX)_] - [ΔCT_(calibratorsample)_] and ΔC_T _= [CT_(targetgene)_] - [CT_(referencegene)_] [24].

### Western blot analysis

Protein extracts were prepared from cells using a protein lysis buffer containing 50 mM Tris-HCl, pH 7.5, 2.0 mM phenylmethylsulfonyl fluoride (PMSF), 5.0 mM iodoacetamide, 5.0 mM ethylene diamine tetraacetic acid (EDTA), 150 mM NaCl, 0.5% nonylphenoxy polyethoxy ethanol (NP-40), and 0.5% nonanoyl-N-methylglucamide (Mega-9). Protease inhibitors leupeptin (2 μg/ml) and pepstatin (1 μg/ml) (Roche, Mannheim, Germany) were added just prior to the addition of lysis buffer to the cells. Protein concentrations in the extracts were quantitated using bicinchoninic acid (BCA) protein assay (Pierce, Rockford, IL). From each extract, 10 to 50 μg of total protein was separated on a 12% SDS-PAGE with a 5% stacking gel. After electrophoresis, proteins were transferred to 0.2 μm PVDF membranes (BioRad, Hercules, CA) using transfer buffer that contained 25 mM Tris-HCl and 700 mM glycine. Membranes were blocked in 7% powdered milk dissolved in 1× TTBS (14 mM Tris, 154 mM NaCl, and 0.1% Tween-20, pH adjusted to 7.5 with HCl) overnight at 4°C. Primary antibodies were incubated for 2 hours at room temperature in 5% powdered milk dissolved in 1× Tween-20 in TTBS. The antibody for the mitochondrial import receptor protein (TOM20) was provided by Dr. Masataka Mori, Kumamoto University School of Medicine, Kumamoto, Japan. Primary antibodies used were: polyclonal rabbit anti-human TOM20 at 1:1,500 dilution, polyclonal rabbit anti-human BNIP3 (BD Pharmingen, San Diego, CA) at 1:8,000 dilution; and polyclonal goat anti-human Cyclin G2 (Santa Cruz Biotechnology) at 1:1,500 dilution. The antigen-antibody complexes were detected using the appropriate secondary antibodies conjugated to horseradish peroxidase (Promega, Madison, WI) dissolved in 1× TTBS with 5% powdered milk and incubated with membranes for 1 hour at room temperature. Membranes were developed using ECL+ (Amersham Pharmacia Biotech, Arlington Heights, IL) and films exposed for appropriate times to detect signal.

### Cell fractionation

The cytosolic and heavy membrane fractions of cells were separated using the ApoAlert Cell Fractionation kit (Clontech Laboratories, Palo Alto, CA) according to manufacturer's instructions. Briefly, cells were mechanically dislodged and collected by centrifugation at 600 × g for 5 minutes at 4°C. The pellets were washed with 1 ml ice-cold wash buffer, and resuspended in 800 μl of ice-cold fractionation buffer. After incubation on ice for 10 minutes, cells were homogenized by 50 strokes using a Dounce homogenizer (pestle B). The homogenates were centrifuged at 700 × g for 10 minutes at 4°C. The supernatant was collected and further centrifuged at 10,000 × g for 25 minutes at 4°C. The resulting supernatant was designated as the cytosol fraction and the pellet (heavy membrane fraction) was resuspended in 100 μl of fractionation buffer mix. Protein concentrations in the extracts were quantitated using bicinchoninic acid (BCA) protein assay (Pierce, Rockford, IL).

## Results

### Genes highly expressed upon Casodex treatment

To analyze the expression of genes that accompany the progression of prostate cancer to the androgen-independent phenotype, we performed three separate cDNA microarray experiments, on androgen-dependent LNCaP-R and androgen-independent LNCaP-UR cells cultured with or without treatment for five weeks in 1 μM Casodex. Hybridizations were performed against a common reference RNA pool so that comparisons across samples and experiments could be made. The commercially available common reference RNA pool contains RNA from ten different cancer cell lines. Of the 2305 genes examined, we found 160 genes whose expression was altered at least 2-fold in the treated cells.

**Figure 1:**
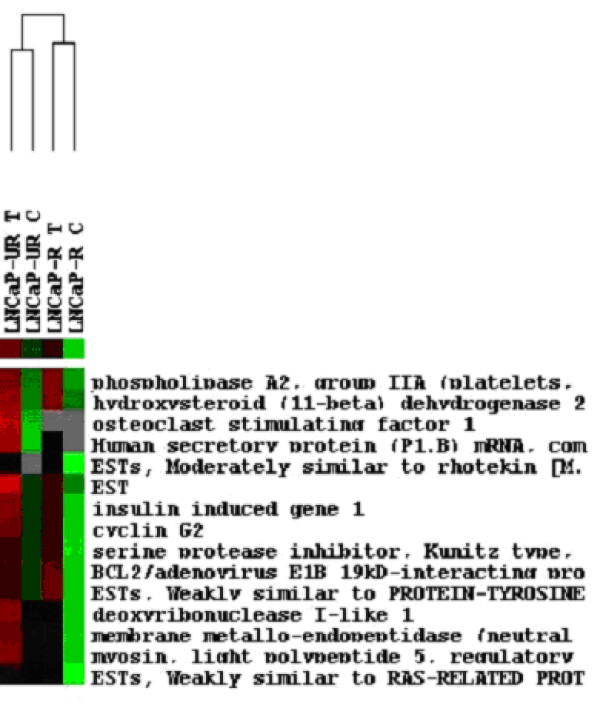
**Cluster diagram of LNCaP cells treated with Casodex**. Clustering was performed as described in Materials and Methods. LNCaP-R C, control. LNCaP-R T, treated 5 weeks in 1 μM Casodex. LNCaP-UR C, control. LNCaP-UR T, treated 5 weeks in 1 μM Casodex. *Columns*, individual samples and *rows*, individual genes. Cells represent the expression level of the indicated gene where green is downregulated, red is up regulated, black is zero, and gray is missing. The correlation in this node is 0.85.

Table 1 shows the list of genes whose expression was significantly increased compared to the reference RNA and Table 2 shows the fold change of these genes in response to treatment with Casodex. These genes were overexpressed in all three hybridizations. After normalization and filtering, a total of 160 genes from the samples were analyzed using hierarchical clustering to show the similarities of expression. The dendogram separated the samples according to overall similarity and shows that control and treated LNCaP-R samples reside on the same branch and control and treated LNCaP-UR samples reside on a parallel branch (Fig. 1). Of the 160 genes clustered were MME, cyclin G2, and BNIP3 all of which showed differential regulation between the LNCaP-R and UR cells and all of which clustered in a same node (Fig. 1). The correlation coefficient of this node was 0.85 which suggests that the expression pattern of these genes was 85% similar.

**Table 1:** Highly expressed Casodex responsive genes in the LNCaP model. Data show fold-increase in gene expression over reference samples. Each datum point represents mean ± SE from 3 independent experiments.

		**LNCaP-R**		**LNCaP-UR**	
**GenBank #**	**Name**	**Control ± SE**	**Treated ± SE**	**Control ± SE**	**Treated ± SE**

AA663884	synaptosomal-associated protein	15.18 ± 3.35	20.67 ± 9.33	20.92 ± 1.07	11.68 ± 0.61
AA700604	sorbitol dehydrogenase	13.73 ± 3.23	19.60 ± 6.50	21.08 ± 1.58	11.08 ± 1.39
T73556	fatty-acid-Coenzyme A ligase	5.11 ± .34	2.45 ± 0.74	3.94 ± 0.46	1.07 ± 0.25
AA028987	EST	5.38 ± 6.95	8.43 ± 5.68	6.22 ± 2.05	11.00 ± 3.74
R98851	membrane metallo-endopeptidase	10.28 ± 5.90	55.90 ± 30.2	79.29 ± 16.62	153.50 ± 56.07
N68465	UDP-N-acteylglucosamine pyrophosphorylase	4.37 ± 3.73	3.99 ± 1.44	12.47 ± 2.03	9.03 ± 2.41
H84113	retinal outer segment membrane 1	8.03 ± 2.06	14.73 ± 4.93	9.79 ± 1.39	7.09 ± 0.65
AA041499	cell division cycle 4-like	11.66 ± 7.07	10.67 ± 1.84	3.47 ± 0.44	3.64 ± 1.14
AA456695	H2B histone family, member Q	4.67 ± 3.02	4.23 ± 1.14	2.56 ± 0.27	4.67 ± 1.13
R82299	S-adenosylmethionine decarboxylase 1	8.35 ± 2.23	14.84 ± 4.06	11.11 ± 2.19	6.98 ± 0.51
AA063521	BCL2/adenovirus E1B 19 kD	3.59 ± 3.34	13.14 ± 3.94	6.76 ± 1.79	12.46 ± 2.32
AA419164	retinoic acid receptor, beta	9.90 ± 3.40	11.14 ± 2.23	7.44 ± 0.98	5.01 ± 0.46
AA459039	serine protease inhibitor, Kunitz type,	2.89 ± 2.90	10.44 ± 1.46	6.28 ± 0.99	9.94 ± 1.44
AA489752	cyclin G2	2.86 ± 2.67	10.97 ± 1.10	6.92 ± 1.62	15.14 ± 4.80
AA412053	CD9 antigen (p24)	4.80 ± 1.05	8.78 ± 2.72	5.44 ± 0.96	6.12 ± 0.84

**Table 2:** Gene expression fold change in the LNCaP model treated with Casodex.

		**Fold Change**	
**GenBank#**	**Name**	**LNCaP-R Control vs. Treated**	**LNCaP-UR Control vs. Treated**

T73556	fatty-acid-Coenzyme A ligase	- 2.09	- 3.68
R98851	membrane metallo-endopeptidase	+ 5.44	+ 1.94
AA063521	BCL2/adenovirus E1B 19 kD	+ 3.66	+ 1.84
AA459039	serine protease inhibitor, Kunitz type,	+ 3.61	+ 1.58
AA489752	cyclin G2	+ 3.84	+ 2.19

(+, fold up regulated and -, fold downregulated)

### Hypoxia regulated gene expression in the LNCaP model treated with Casodex

**Figure 2:**
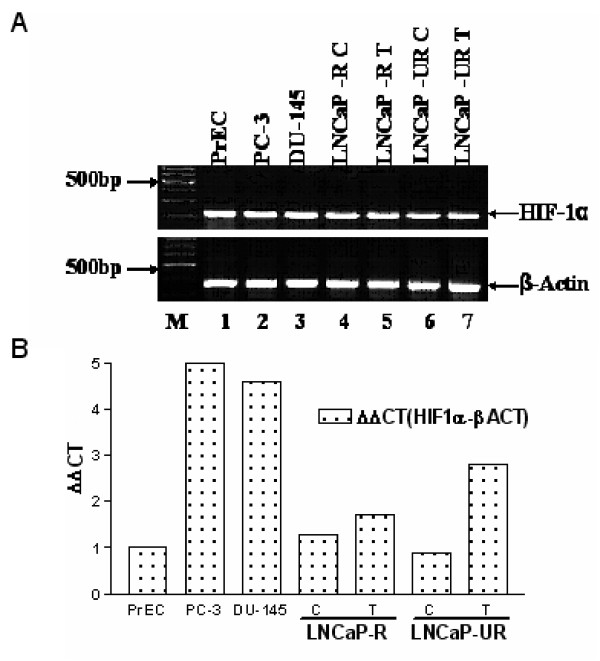
**A. RT-PCR of HIF-1α**. RT-PCR was performed as described in Materials and Methods and shows the presence of HIF-1α (upper panel) and β-actin as internal control (lower panel). Lane 1, normal prostate (PrEC). Lanes 2 and 3, androgen-independent prostate cell lines PC3 and DU145, respectively. Lane 4, LNCaP-R control. Lane 5, LNCaP-R treated 5 weeks in 1 μM Casodex. Lane 6, LNCaP-UR control. Lane 7, LNCaP-UR treated 5 weeks in 1 μM Casodex. B. Quantitative real time PCR for HIF-1α expression was perfomed as described in materials and methods. CT values was determined using the formula ΔΔCT = [ΔCT_(sampleX)_] - [ΔCT_(calibratorsample)_] and ΔC_T _= [CT_(targetgene)_] - [CT_(referencegene)_]. The graph was drawn using Graph Pad Prism 3.0.

HIF-1 is a transcriptional activator involved in oxygen homeostasis in cells [25]. In tumors where hypoxic conditions are common, HIF-1 levels are increased and stimulate gene expression that promotes angiogenesis [26]. HIF-1α is the inducibly expressed subunit that forms a heterodimer with the constitutively expressed HIF-1β. HIF-1α has been implicated in androgen-independent prostate cancer [13] and is thought to mediate angiogenesis in tumors by transcriptional up regulation of vascular endothelial growth factor [27]. BNIP3 was shown to be regulated by HIF-1α [28,29] which led us to examine whether the high levels of BNIP3 detected in our hybridizations were accompanied by expression of HIF-1α. Figure 2 (panel A) shows the results of an RT-PCR and reveals the expression of HIF-1α in normal human prostate epithelial cells, PC3 and DU145 cells, and the LNCaP model. Using RT-PCR, we did not observe significant differences in the intensity of the PCR band between different cell lines and upon treatment with Casodex. To more quantitatively assess the message level, we performed a quantitative Real Time PCR analysis of HIF-1α along with β-actin as an internal control. The data presented in Figure 2 (panel B) shows a clear up regulation of HIF-1α expression in the androgen-independent PC3 and DU145 cells when compared to the low levels expressed in normal prostate epithelial cells (PrEC). In the LNCaP model, low levels of HIF-1α are expressed in the untreated LNCaP-R and LNCaP-UR cells. However, upon Casodex treatment, both LNCaP-R and UR cells show increased expression of HIF-1α which more closely resembles the expression pattern observed in the androgen-independent PC-3 and DU-145 cells.

### Verification of MME, Cyclin G2, and BNIP3 message in the LNCaP model

**Figure 3:**
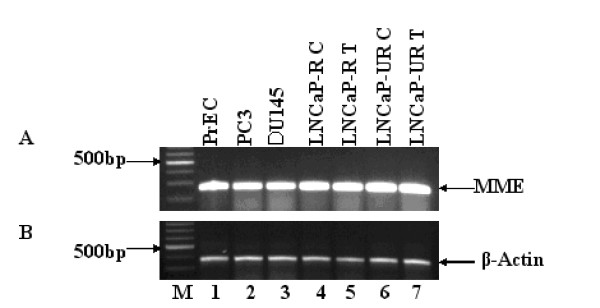
**RT-PCR of MME**. RT-PCR was performed as described in Materials and Methods and confirms the presence of MME. A single band at 268 bp (black arrow) indicates MME message expression. Lane 1, normal prostate (PrEC). Lanes 2 and 3, androgen-independent prostate cell lines PC3 and DU145, respectively. Lane 4, LNCaP-R control. Lane 5, LNCaP-R treated 5 weeks in 1 μM Casodex. Lane 6, LNCaP-UR control. Lane 7, LNCaP-UR treated 5 weeks in 1 μM Casodex. β-actin (bottom panel) was included as a control.

MME is a neutral endopeptidase expressed on the cell surface and catalyzes the degradation of biologically active neuropeptides [30]. Prostate epithelial cells express MME, including the LNCaP prostate cancer cell line, and there is evidence that the presence of MME on the surface of prostate cancer cells promotes metastasis [31,32]. In contrast, other reports suggest that MME can inhibit prostate cancer cell growth [33] and can inhibit prostate cancer cell migration [34] and loss of MME expression can contribute to the progression to androgen-independence [35]. MME was shown to be up regulated at the transcriptional level by androgen in LNCaP prostate cancer cells [36]. RT-PCR was performed to verify message expression of MME. Figure 3 shows the results of RT-PCR of MME in normal human prostate epithelial cells, PC3 and DU145 cells, and the LNCaP model control and treated with Casodex. The data show a single product of expected size (268 bp) in all samples tested. As a normalization control, we included β-actin in parallel experiments (Figure 3, panel B). MME protein was reported to be undetectable in PC3 and DU145 cells [32]. However, our results show that MME message was expressed in PC3, DU145 and LNCaP cells.

**Figure 4:**
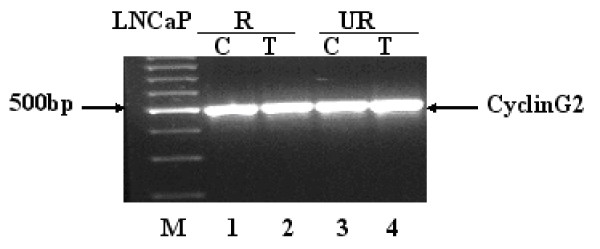
**RT-PCR of Cyclin G2**. RT-PCR was performed as described in Materials and Methods and confirms the presence of Cyclin G2 message expression in LNCaP cells control or treated with 1 μM Casodex for 5 weeks. Cyclin G2 message is indicated (black arrow) at 502 bp. Lane 1, LNCaP-R control. Lane 2, LNCaP-R treated 5 weeks in 1 μM Casodex. Lane 3, LNCaP-UR control. Lane 4, LNCaP-UR treated 5 weeks in 1 μM Casodex.

**Figure 5:**
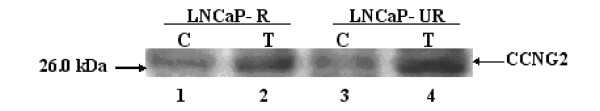
**Western blot of Cyclin G2**. Western blot was performed as described in Materials and Methods and shows the presence of Cyclin G2 (CCNG2) protein in LNCaP cells control or treated with 1 μM Casodex for 5 weeks. Lane 1, LNCaP-R control. Lane 2, LNCaP-R treated 5 weeks in 1 μM Casodex. Lane 3, LNCaP-UR control. Lane 4, LNCaP-UR treated 5 weeks in 1 μM Casodex.

Cyclin G2 is a cell cycle inhibitor [37] whose expression is induced during apoptosis. Cyclin G2 is expressed at varying levels through the cell cycle and was shown to be present in cerebellum, thymus, spleen, kidney and prostate tissue [38]. Interestingly, cyclin G2 was recently shown to be induced by hypoxia [39]. Figure 4 verifies cyclin G2 message expression (product at 502 bp) in the LNCaP cells treated or untreated with Casodex. RT-PCR confirmed the presence of cyclin G2 in control and treated LNCaP cells. However, western blot analysis showed that Casodex-treated cells overexpressed cyclin G2 protein (Figure 5). Figure 5 shows protein levels of cyclin G2 in LNCaP cells treated or untreated with Casodex. Cyclin G2 protein levels appear to be up regulated upon treatment with Casodex in both the LNCaP-R (lane 2) and LNCaP-UR (lane 4) cells. These results are consistent with cyclin G2 message up regulation indicated in microarray analysis (Table 2).

**Figure 6:**
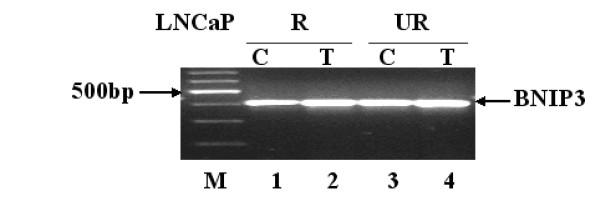
**RT-PCR of BNIP3**. RT-PCR was performed as described in Materials and Methods and confirms the presence of BNIP3 message expression in LNCaP cells control or treated with 1 μM Casodex for 5 weeks. BNIP3 message expression is indicated (black arrow) at 411 bp. Lane 1, LNCaP-R control. Lane 2, LNCaP-R treated 5 weeks in 1 μM Casodex. Lane 3, LNCaP-UR control. Lane 4, LNCaP-UR treated 5 weeks in 1 μM Casodex.

BNIP3 is a proapoptotic Bcl-2 family member localized to the mitochondrial membrane and was shown to interact with antiapoptotic family members to promote apoptosis [40]. The transmembrane region of BNIP3 localizes this protein to the mitochondrial membrane and it was shown that deletion mutants lacking the transmembrane region were unable to induce apoptosis [40]. BNIP3 was shown induce apoptosis in hypoxic cardiac cells. [41-43] and is itself induced by hypoxia in tumors [29]. Figure 6 verifies by RT-PCR BNIP3 message expression (411 bp) in the LNCaP model control and treated with Casodex. RT-PCR's were repeated for each MME, cyclin G2, and BNIP3 biological replicates and similar results were observed (data not shown).

### BNIP3 in the LNCaP model treated with Casodex

**Figure 7:**
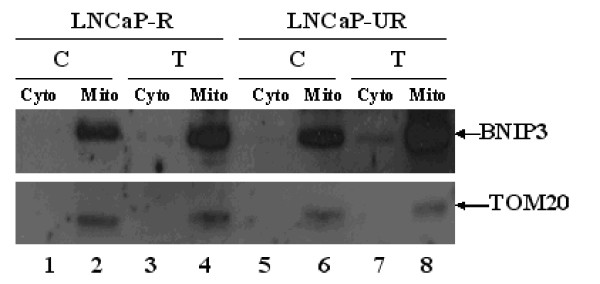
**Cell fractionation and western blot of LNCaP cells treated with Casodex**. Cell fractionation of cytosolic (Cyto) and mitochondrial (Mito) fractions and western blot was performed as described in Materials and Methods. LNCaP cells either treated with 1 μM Casodex for 5 weeks or control were fractionated and subjected to western blot and show that LNCaP-UR cells overexpress BNIP3 which is predominately localized in the mitochondrial fraction. Lane 1, LNCaP-R control cytosolic fraction. Lane 2, LNCaP-R control mitochondrial fraction. Lane 3, LNCaP-R treated with 1 μM Casodex for 5 weeks, cytosolic fraction. Lane 4, LNCaP-R treated with 1 μM Casodex for 5 weeks, mitochondrial fraction. Lane 5, LNCaP-UR control cytosolic fraction. Lane 6, LNCaP-UR control mitochondrial fraction. Lane 7, LNCaP-UR treated with 1 μM Casodex for 5 weeks, cytosolic fraction. Lane 8, LNCaP-UR treated with 1 μM Casodex for 5 weeks, mitochondrial fraction. TOM20, a mitochondrial integral membrane protein, (bottom panel) was included as a control.

In order to assess the significance of BNIP3 message up regulation in the LNCaP model, we performed western blots to determine the subcellular localization of the protein. Cytosolic and mitochondrial fractions were subjected to western blot analysis and TOM20, an integral mitochondrial membrane protein [44], was included as an internal control. Figure 7 shows that BNIP3 was detected in the mitochondrial fraction of both LNCaP-R and UR cells (lanes 2, 4, 6, and 8), and BNIP3 is overexpressed in the Casodex-treated LNCaP-R and LNCaP-UR cells.

### Expression of other hypoxia-related genes

**Figure 8:**
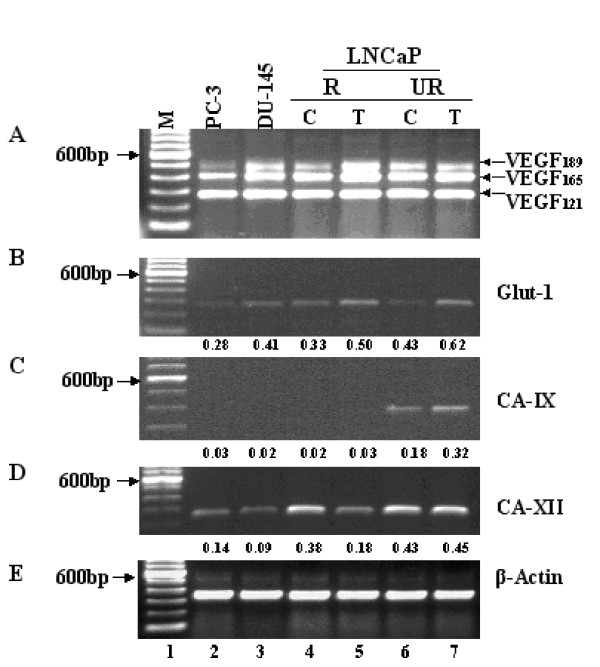
**RT-PCR of HIF-1 induced genes**. LNCaP cells were treated with 1μM Casodex for 5 weeks. RNA was isolated from both treated and untreated cells as described in Materials and Methods. Expression of VEGF isoforms (panel A), Glut-1 (panel B), CA-IX (panel C), CA-XII (panel D) and β-Actin (panel E) were examined by RT-PCR. In each panel, the cell lines shown are PC-3 (lane 2), DU-145 (lane 3) LNCaP-RC (lane 4), LNCaP-RT (lane 5), LNCaP-URC (lane 6) and LNCaP-URT (lane 7). The 100 bp DNA ladder (lane1) is included as a size marker. PCR amplification was performed as described under Materials and Methods.

In order to determine if other hypoxia-related genes were being regulated in the LNCaP model treated with Casodex, we measured mRNA expression levels of vascular endothelial growth factor (VEGF), glucose transporter-1 (Glut-1), and carbonic anhydrases 9 and 12 (CAIX and CAXII). VEGF isoforms 189, 165, and 121 are shown in Fig. 8, panel A. We did not detect a band representative of VEGF isoform 145. Isoform 121 showed relatively equal expression in all cells tested however, isoform 165 appeared upregulated in LNCaP-R cells after five weeks treatment with Casodex (Fig. 8 panel A. lane 5). Isoform 189 appeared upregulated in DU145 cells (lane 2) compared to PC3 cells (lane 1) and increased in LNCaP-R and UR cells after treatment with Casodex (lanes 5 and 7, respectively). Increased glucose uptake, mediated by Glut-1, is thought to protect cells from apoptosis in hypoxic conditions [45]. Expression levels of Glut-1 are shown in Fig. 8 panel B and shows basal levels of Glut-1 in PC3 and DU145 cells (lanes 1 and 3). Treatment of LNCaP-R and UR cells with Casodex resulted in an upregulation of Glut-1 message (lanes 5 and 7, respectively). CAIX and XII are inducible by HIF-1α [39] and were shown to be expressed at very low levels in PC3 cells and negative for expression in DU145 cells [46]. Fig. 8 panel C shows CAIX expression is barely detectable in PC3, DU145, LNCaP-R control and treated with Casodex (lanes 2 through 5). LNCaP-UR cells showed an approximate two-fold increase in CAIX expression after treatment with Casodex (lanes 6 and 7). CAXII expression was detected in both PC3 and DU145 cells (Fig. 8, panel D, lanes 1 and 2), however, in LNCaP-R cells treated with Casodex, the levels appeared to decrease approximately two-fold (lanes 4 and 5). LNCaP-UR cells showed approximate equal expression of CAXII after treatment with Casodex (lanes 6 and 7).

## Discussion

In this study, cDNA microarray analysis was performed to identify genes involved in prostate cancer cell response to chronic treatment with Casodex. The LNCaP prostate cancer progression model was used due to its ability to progress from an androgen-dependent to an androgen-independent phenotype [17]. With this model, it was possible to measure changes in gene expression that accompanied progression from androgen-dependence to androgen-independence in a similar genetic background in response to chronic treatment with Casodex. Since patients with both androgen-dependent and androgen-independent prostate cancer are treated with androgen-ablation therapy, it is important to understand what changes in gene expression occur in both cases in order to identify potential therapeutic targets for more specific intervention independent of androgen-signaling status.

The treatment time course of LNCaP in Casodex containing media was based on pilot studies which showed no change in the antiapoptotic protein Bcl-2 after growth of the cells for three weeks. After five weeks in Casodex containing media, there was an apparent decrease in Bcl-2 protein and we measured a decrease in apoptosis [18]. We surmised from these studies that the changes occurring in LNCaP cells resulted after extended exposure to Casodex and therefore, set the time course of five weeks treatment.

The three independent microarray analyses consistently showed a set of highly expressed genes (Table 1). Given that the reference RNA contained a set of 10-pooled cancer cell lines, we termed these highly expressed genes as "prostate cancer signature genes". We chose to further examine expression of MME, cyclin G2, and BNIP3 since these genes were significantly up regulated in the LNCaP model upon treatment with Casodex (Table 2) and these genes are each involved in hypoxia-response.

Several genes showed downregulated expression in response to Casodex treatment in LNCaP-UR cells (Table 1) however, only fatty-acid-Coenzyme A ligase showed downregulated expression in both LNCaP-R and UR cells (Table 2). Although we did not further investigate this downregulation, it is possible that hypoxic conditions resulted in fatty-acid metabolism alterations which caused the downregulation of this ligase.

Clustering of the 160 genes showed that MME, cyclin G2, and BNIP3 were similarly expressed (Fig. 1) and clustered in a similar node (correlation = 0.85) which supports the notion that a hypoxic response is a predominant phenomena in LNCaP cells response to treatment with Casodex. The dendogram in Figure 1 revealed the similarities and differences between representative cell lines in our LNCaP model. Control and treated LNCaP-R samples clustered in the same branch and in parallel, control and treated LNCaP-UR samples clustered in the same branch (Fig.1). Since LNCaP-R and LNCaP-UR cells differ in terms of growth, morphology, response to androgen, and sensitivity to apoptosis [16], it was expected the two would cluster in separate branches. These data demonstrate the distinct genetic expression profiles largely due to the androgen-dependent and androgen-independent difference between LNCaP-R and LNCaP-UR cells.

We probed for HIF-1α expression by RT-PCR. The results showed the presence of HIF-1α at the expected molecular weight in all cells. Quantitative PCR analysis indicated up regulation of HIF-1α in Casodex treated LNCaP-R and LNCaP-UR cells (Figure 2, panel B). This result is consistent with up regulation of hypoxia-related genes upon treatment with Casodex (Tables IA and IB). Further, up regulation of HIF-1α in the Casodex-treated cells is similar to the high levels of HIF-1α in the androgen-independent PC3 and DU145 cells, indicating that Casodex treatment may drive the LNCaP cells towards androgen-independence, consistent with our previous observations [18].

We verified the identity and expression of some of the genes identified by microarray analysis and relevant to hypoxia. MME, cyclin G2, and BNIP3 message identity and expression were confirmed by RT-PCR analysis (Figs. 3, 4, and 6, respectively). MME has been implicated in prostate cancer and though the data are conflicting on whether this gene promotes or protects against the progression, our data suggest a role for this gene in prostate cancer progression to androgen-independence. Though others report that MME was not detectable in PC3 and DU145 cells [29]. This disparity may be due to subtle differences in culturing techniques or PCR conditions between our laboratory and others working with these cells. In the LNCaP-R cells, MME expression was up regulated over 5-fold and in LNCaP-UR cells MME was up regulated nearly 2-fold in response to Casodex (Table 2). In context, MME was up regulated over 150-fold in the LNCaP-UR cells compared to the reference RNA (Table 1) which strongly suggests this gene plays a role in androgen-independent prostate cancer. Precisely what function MME plays in the LNCaP model is currently under investigation.

Cyclin G2 was shown to inhibit cell cycle progression and promote apoptosis [37]. Our data showed an increase in cyclin G2 message (Table 2) and protein expression (Fig. 5, lanes 2 and 4) in the LNCaP model upon treatment with Casodex. Aside from the known induction of cyclin G2 by HIF-1α [39] the increased expression of cyclin G2 in our Casodex treated samples could also be due to the long duration of time the cells spend in the culture flask. The experiment warrants that Casodex treatment begins after the cells have come to approximately 70% confluence. At this density, there is a short time period after the addition of media containing Casodex of sustained logarithmic growth after which the cells appear to cease growth before the 5 weeks treatment has ended (visual inspection, data not shown). It is possible that cyclin G2 is involved in the growth arrest observed in the LNCaP model during treatment with Casodex.

BNIP3 is a proapoptotic Bcl-2 family member and as such, its up regulation and overexpression would predict induction of apoptosis. We have previously shown that in LNCaP cells treated with 1 μM Casodex for 5 weeks then treated with 5 nM docetaxel, the cells pretreated for 5 weeks in Casodex exhibited an increased resistance to the induction of apoptosis by docetaxel when compared to cells not pretreated with Casodex but treated with docetaxel [18]. We addressed the possibility that BNIP3 protein may be missorted or mutated. It was shown that C-terminal deletion mutants of BNIP3 lost the ability to induce apoptosis [40]. We did detect the presence of BNIP3 in the mitochondrial membrane fraction of cells suggesting that BNIP3 is localized correctly to the mitochondrial membrane presumably via its C-terminal transmembrane region. Determining why the increased levels of BNIP3 do not induce apoptosis in the LNCaP model is currently under investigation.

Expression of VEGF isoforms 165 and 121 showed similar levels in LNCaP-R and UR cells treated with Casodex. However, the 189 isoform appeared to be upregulated upon treatment with Casodex. The 189 isoform may be preferentially induced in response to Casodex in the LNCaP model. The absence of VEGF isoform 145 in all cells tested suggest either that it is not a preferred expressed isoform in prostate cancer cells or that our PCR cycling conditions did not favor its expression though this latter possibility is unlikely since the primers used were designed to amplify all four splice variants. Glut-1 expression levels showed upregulation in the LNCaP model after treatment with Casodex suggesting that the cells were responding to Casodex treatment by presumably increasing glucose uptake to protect against hypoxia-induced damage. CAIX and XII showed variable expression (Fig. 8. panels C and D) with CAIX detected only in the LNCaP-UR cells. CAXII showed variable expression in all cells and was downregulated in LNCaP-R cells treated with Casodex (Fig. 8 panel D, lane 5) but appeared unchanged in LNCaP-UR cells after treatment (lanes 6 and 7). These data suggests that CAIX and XII may not be major contributors in LNCaP cells response to Casodex treatment.

It was recently shown, using the LNCaP prostate cancer cell line, that one of the mechanisms of Casodex in prostate cancer was the assembly of an inactive transcriptional complex on androgen response elements in the DNA suggesting that altered coactivator and/or corepressor genes may be responsible for androgen-independent progression [47]. Our data showing differential expression of hypoxia related genes up regulated upon treatment with Casodex are supported by this finding.

## Conclusion

We conclude from these studies that hypoxia plays a critical role in the progression of prostate cancer to the androgen-independent phenotype and furthermore, identify induction of a hypoxic response as one of the mechanisms of Casodex in prostate cancer.

## Competing interests

The author(s) declare that they have no competing interests.

## Authors' contributions

CAR and VKG contributed equally to the work. CAR carried out the cell culture, treatment and microarray studies. VKG performed the immunoblot analyses. JDE performed the microarray analysis and assisted in data analysis. JKV conceived of the study, participated in its design and analysis, and direct the manuscript. All authors read and approved the final manuscript.

## Pre-publication history

The pre-publication history for this paper can be accessed here:


